# THE LIMITS OF FORTITUDE: PRINCIPLES FOR ADVANCING EQUITABLE AGING POLICY

**DOI:** 10.1093/geroni/igae098.2093

**Published:** 2024-12-31

**Authors:** Jane Lowers

**Affiliations:** Emory University, Atlanta, Georgia, United States

## Abstract

Fortitude – an individual’s ability to endure difficulty or adversity – is an implicit premise in U.S. aging policies, which require individuals and families to shoulder the financial, physical, and emotional costs of aging. Emphasizing fortitude in any discussion of aging obscures the assumption that it works best when accompanied by a lifetime accumulation of wealth and other tangible and intangible supports. When systemic supports for aging are fractured and inadequate, no amount of individual fortitude can adequately bolster older adults challenged by fixed incomes, decreased availability of family caregivers, and lengthening years with chronic and progressive illness. Using examples of adults aging solo with dementia, this presentation will propose alternatives to fortitude as organizing principles for rethinking U.S. aging policy.

